# Use of ^177^Lu prostate-specific membrane antigen therapy in metastatic castration-resistant prostate cancer: Joint statement of the Austrian Society of Nuclear Medicine and Theranostics and the Austrian Society of Urology and Andrology

**DOI:** 10.1007/s00508-025-02544-4

**Published:** 2025-06-04

**Authors:** Siroos Mirzaei, Gregor Schweighofer-Zwink, Heidemarie Ofner, Jasmin Bektic, Harun Fajkovic, Eisabeth von Guggenberg, Anton Ponholzer, Gero Kramer, Lukas Lusuardi, Michael Gabriel, Tatjana Traub-Weidinger, Marcus Hacker, Shahrokh F. Shariat

**Affiliations:** 1 Department of Nuclear Medicine with PET Center, Clinic Ottakring, Vienna, Austria; 2 Department of Nuclear Medicine and Endocrinology, Paracelsus Medical University Salzburg, Salzburg, Austria; 3 Department of Urology, Medical University of Vienna, Währinger Gürtel 18–20, 1090 Vienna, Austria; 4 Department of Urology, Comprehensive Cancer Center, Medical University of Vienna, Vienna, Austria; 5 Department of Urology, Medical University of Innsbruck, Innsbruck, Austria; 6 Department of Urology, Medical University of St. Pölten, St. Pölten, Austria; 7 Department of Nuclear Medicine, Medical University of Innsbruck, Innsbruck, Austria; 8 Department of Urology, University Hospital Salzburg, Paracelsus Medical University, Salzburg, Austria; 9 Nuclear Medicine and Endocrinology, Kepler University Hospital, Linz, Austria; 10 Department of Diagnostic and Therapeutic Nuclear Medicine, Clinic Donaustadt, Vienna Health Care Group, Vienna, Austria; 11 Department of Biomedical Imaging and Image-Guided Therapy, Medical University of Vienna, Vienna, Austria; 12 Department of Urology, University of Texas Southwestern, Dallas, TX USA; 13 Hourani Center for Applied Scientific Research, AI-Ahliyya Amman University, Amman, Jordan; 14 Research Center for Evidence Medicine, Urology Department, Tabriz University of Medical Science, Tabriz, Iran; 15 Department of Urology, Weill Cornell Medical College, New York, NY USA

**Keywords:** Prostate Cancer, ^177^LU-PSMA, Radioligand therapy, Theranostic

## Abstract

Prostate cancer (PCa) remains a leading cause of cancer-related morbidity and mortality in men. Over the past decades, the incidence has risen in all age groups. Prostate-specific membrane antigen (PSMA)-based theranostics, which involve the use of PSMA-targeting radiopharmaceuticals for both diagnostic imaging and therapeutic applications, have become integral in the management of patients with metastatic castration-resistant prostate cancer (mCRPC). The field has witnessed a substantial and continuously expanding body of scientific literature, encompassing case reports, original research, systematic reviews and also clinical guidelines. The objective of this joint statement is to enhance awareness of PSMA theranostics and underscore their critical role in contemporary precision oncology. We aim to provide a comprehensive yet practical perspective on the current landscape of PSMA-based diagnostic and therapeutic modalities, offering a consensus-driven approach to their clinical application.

## Aim

Prostate-specific membrane antigen (PSMA)-based theranostics, which involve the use of PSMA-targeting radiopharmaceuticals for both diagnostic imaging and therapeutic applications, have become integral in the management of patients with metastatic castration-resistant prostate cancer (mCRPC). The field has witnessed a substantial and continuously expanding body of scientific literature, encompassing case reports, original research, systematic reviews but also prospective randomized trials and clinical guidelines.

The objective of this joint statement is to enhance awareness of PSMA theranostics and underscore their critical role in contemporary precision oncology. We aim to provide a comprehensive yet practical perspective on the current landscape of PSMA-based diagnostic and therapeutic modalities, offering a consensus-driven approach to their clinical application. Rather than serving as a traditional review, this statement seeks to bridge the rapidly accumulating scientific evidence with existing guideline recommendations, facilitating their effective translation into clinical practice.

## Introduction

Prostate cancer (PCa) remains a leading cause of cancer-related morbidity and mortality in men, with an estimated incidence of 1.4 million cases worldwide in 2020 with 375,000 deaths from the disease in the same year [1]. Over the past 20 years, incidence has risen in all age groups. The Lancet Commission on prostate cancer expects a surge in cases globally with up to 2.9 million new cases in 2040 [2]. Although synchronous metastatic disease is rare (10–15%) at initial diagnosis, up to 20–40% patients suffer from relapse of disease.

Metastatic castration-resistant prostate cancer (mCRPC) represents an advanced stage of prostate cancer, characterized by disease progression during androgen deprivation therapy (ADT). It arises due to adaptive resistance mechanisms, including androgen receptor (AR) pathway alterations and leads to changes in tumor biology and heterogeneity. mCRPC is associated with poor prognosis, necessitating advanced therapeutic strategies [3]. In recent years, advancements in the diagnosis and treatment of PCa have been made through developing innovative and personalized treatment approaches, including theranostics. PSMA is highly expressed on PCa cells and high expression was related to high Gleason scores and International Society of Urological Pathology** (**ISUP) grades. PSMA, thus, was identified as the ideal target for radioligand therapy (RLT) with PSMA agents, delivering beta- or alpha-radiation targeted to PCa cells, mainly sparing healthy tissue [4, 5].

In light of the growing burden of PCa, the development and accessibility of advanced personalized treatments becomes increasingly crucial in addressing this major public health challenge.

## Spectrum of disease and therapeutic implications

Prostate cancer encompasses a heterogeneous spectrum of disease states, each requiring a tailored therapeutic approach. In cases where the disease remains confined to the prostate gland, radical prostatectomy or radiation therapy serve as potentially curative interventions, particularly for patients with intermediate- to high-risk features, who face an elevated risk of metastatic progression. For patients with low-risk disease, active surveillance represents a clinically sound strategy, minimizing overtreatment and reducing the burden of unnecessary adverse effects while preserving quality of life [6, 7].

In patients with metastatic castration sensitive prostate cancer (mCSPC) current guidelines recommend dual or triple combination therapy with ADT plus a novel hormonal therapy (ARTA = androgen receptor targeted agents) and additionally docetaxel in the triple therapy setting. Treatment selection is based on patient characteristics and prognostic factors, including timing of metastases (synchronous vs. metachronous) and metastatic burden (high vs. low-volume, according to CHAARTED criteria). Prognoses vary significantly: patients with low-volume, metachronous mCSPC have the most favorable outcomes, with a 5-year overall survival (OS) of 70–75%, while those with high-volume, synchronous mCSPC face a poorer prognosis, with a 5-year OS of 20–30%, necessitating more intensive, personalized treatment approaches [6, 7].

Despite effective treatment options for mCSPC, most patients progress to the metastatic castration resistant stage of disease. Treatment aims to prolong survival, manage symptoms, and maintain quality of life through a range of therapeutic options tailored to patient characteristics and molecular profiles. Among the systemic treatment options are again ARTAs but also taxane-based chemotherapy (docetaxel and/or cabazitaxel) or poly(ADP-ribose)-polymerase (PARP) inhibitors either as monotherapy or combination therapy, especially in patients with, but also without HRR gene mutations [8, 9]. Of importance, radionuclide therapies including radium-223 (for bone only metastases) and PSMA-targeted RLT have demonstrated survival benefits in this stage of disease [10, 11].

In summary, the therapeutic landscape for metastatic PCa has evolved significantly over the past 5 years, emphasizing the importance of a personalized treatment approach to achieve optimal patient outcomes.

## PSMA expression, imaging and targeted therapy

In the last decade PSMA has proven its pivotal role as diagnostic as well as therapeutic target in PCa. This is attributed to several aspects of this transmembrane glycoprotein.

- PSMA is overexpressed in almost all PCa lesions as well as their lymph node and bone metastasis throughout different disease stages as compared to benign prostatic tissue [12].
- The expression of PSMA in PCa cells may even exceed the 100- to 1000-fold as compared to its expression in benign tissue [13].
- The expression regularly increases even further in PCa cells reaching a castration resistant state [14].
- If an antibody or a PSMA-inhibitor binds to the extracellular domain of PSMA, the whole complex is internalized into the cell leading to an accumulation of the binding molecule [15].
- Both the significant overexpression of PSMA in PCa as well as the intracellular accumulation of ligand molecules translate into an impressive tumor to background ratio, when using PSMA antagonists as imaging agents in nuclear medicine.

**Fig. 1:**
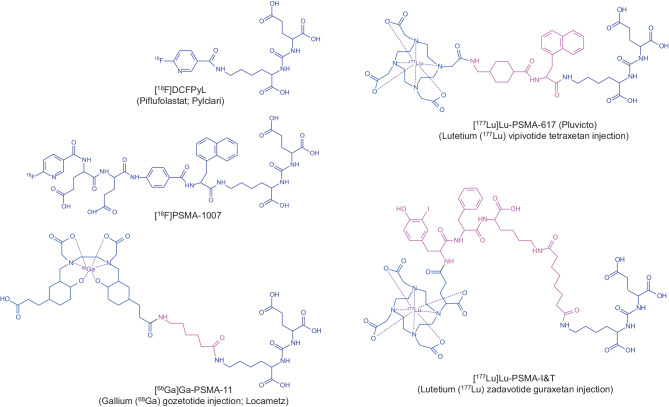
Chemical structures of various radiolabeled prostate-specific membrane antigen (PSMA) inhibitors which are used for diagnostic and therapeutic purposes

In recent years different PET tracers have been introduced for staging of patients with advanced local disease or for detection of recurrent disease in cases with biochemical recurrence, including different PSMA ligands labelled with gallium-68 (e.g., ^68^Ga-PSMA-11), fluorine-18 (^18^F‑DCFPyl, ^18^F‑PSMA-1007), and even copper-64 (e.g., ^64^Cu-PSMA-I&T; [11, 16]). Figure 1 illustrates the chemical structures of different radiopharmaceuticals used for diagnostic and therapeutic purposes. The radiopharmaceuticals are provided either from different commercial suppliers or available via in house radiopharmacy production according to European Pharmacopoeia monographs (No. 3170 Ph.Eur.11.7).

Furthermore, PSMA-positron emission tomography/computed tomography (PET/CT) has been used for the inclusion and monitoring of patients for RLT in mCRPC patients [23].

In daily routine, PSMA-PET images are interpreted qualitatively in comparison to lesion background, hepatic, parotideal and blood pool activity, but some centers are also reporting quantitative SUV values. The so-called PROMISE criteria help to form a standardized set of interpretation and reporting system aiding to minimize interreader as well as interinstitutional differences [24].

Due to its expression and internalization behavior, as described above, PSMA forms an ideal basis for a targeted therapy using radionuclides like a beta minus emitter, e.g. lutetium-177.

## Radiopharmaceuticals for PSMA therapy

RLT with two different PSMA ligands labelled with the beta emitter lutetium-177, namely ^177^Lu-PSMA-I&T (I&T: imaging and therapy) and ^177^Lu-PSMA-617 showed favorable safety in mCRPC patients. Both radiopharmaceuticals showed highest absorbed doses among healthy organs in the lacrimal glands and parotid glands, but this did not result in significant clinical consequences. ^177^Lu-PSMA-617 had a higher absorbed dose to the whole body and lacrimal glands, but a lower renal dose than ^177^Lu-PSMA-I&T. The mean absorbed tumor doses were comparable for ^177^Lu-PSMA-I&T and ^177^Lu-PSMA-617. Dosimetry parameters varied widely between patients. Therefore, individual patient-specific dosimetry was recommended [25].

For dosimetric purposes, usually after the first administration, a medical physicist must be available for consultation.

## Current guideline recommendations on ^177^Lu-PSMA-617 in PCa

Both the efficiency and the tolerability led to the endorsement of ^177^Lu-PSMA-RLT in major international guidelines. Table 1 gives a brief overview about current guideline recommendations.

**Table 1:** EAU, ESMO, NCCN, AUA and EANM/SNMMI treatment recommendations summarized

Guideline	Recommendation
*EAU* Guidelines on Prostate Cancer 2024 [26]	Offer ^177^Lu-PSMA-617 to pre-treated mCRPC patients with ≥1 highly PSMA expressing metastatic lesion
*ESMO* updated treatment recommendations for prostate cancer 2023 [27]	In mCRPC patients, who have received an ARTA and docetaxel, the following treatments should be considered:
	^177^Lu-PSMA-617 (with cancer expressing PSMA on PSMA-PET and without PSMA non-expressing lesions)
	Cabazitaxel
*NCCN* Guidelines Recommendations on Systemic Therapy for M1 CRPC AdenoCa 2023 [28]	In mCRPC, after progression on docetaxel and an ARTA, useful in certain circumstances:
	^177^Lu-PSMA-617 (≥ 1 PSMA-positive lesion and/or metastatic disease that is predominately PSMA-positive and with no dominant PSMA-negative metastatic lesions)
*AUA* Guidelines on Advanced Prostate Cancer, Guideline Statement 32 Amended 2023 [29]	^177^Lu-PSMA-617 should be offered in patients with progressive mCRPC, previously treated with docetaxel and an ARTA, with a positive PSMA-PET imaging
*EANM/SNMMI*	In mCRPC PSMA-positive patients, who progressed at least after an ARTA or alternative treatment options have been exhausted or are contraindicated on an individual patient basis
*German Guidelines on Prostate Cancer* *(S3 Leitlinie Prostatakarzinom)* 2024	mCRPC patients with progressive disease, in a good general condition, after docetaxel and one ARTA should be offered:
	Cabazitaxel
	^177^Lutetium-PSMA-617 (^177^Lu vipivotide tetraxetan)

*ARTA* androgen receptor targeted agents, *mCRPC* metastatic castration-resistant prostate cancer, *PSMA* Prostate-specific membrane antigen

## ^177^Lu-PSMA-RLT treatment evidence

Based on the phase III randomized controlled VISION trial, ^177^Lu-PSMA-617 was approved by the US Food and Drug Administration (FDA) and European Medicines Agency (EMA) in 2022. The two alternate primary endpoints Radiographic Progression-Free Survival (rPF)S and OS were positive with a prolongation of overall survival of 4 months (median 15.3 vs. 11.3 months; hazard ratio (HR) for death 0.62; 95% confidence interval [CI], 0.52–0.74; *p* < 0.001). mCRPC patients, who had undergone prior ARTA and taxane-based chemotherapy treatment, were included [10]. The phase II TheraP trial further supported the efficacy of ^177^Lu-PSMA-617, demonstrating a higher PSA response rate compared to cabazitaxel in mCRPC patients [30].

Expanding its use to earlier disease stages, the PSMAfore trial included taxane-naïve mCRPC patients, with a trial comparator arm of an ARTA switch. The primary endpoint rPFS was nearly doubled (11.6 vs. 5.6 months), yet OS did not reach statistical significance in the trial. This might be due to the study design, allowing a crossover from patients in the control arm into the investigational arm disease progression, highly probably confounding the analysis of OS [31].

Moving to even earlier disease stages, UpFrontPSMA investigated a sequential treatment with ^177^Lu-PSMA-617 followed by docetaxel in mHSPC patients, leading to significantly higher undetectable PSA rates [32]. PSMAddition similarly has included mHSPC patients with two study arms: ^177^Lu-PSMA-617 + standard of care (SOC) vs. SOC alone—the final analysis of this trial is awaited. Finally, the LuTectomy trial included high risk localized PCa patients, who received two cycles of neoadjuvant ^177^Lu-PSMA-RLT before radical prostatectomy [33]. Nevertheless, ^177^Lu-PSMA-RLT is not yet approved in earlier disease stages than in mCRPC stages and the evidence in a perioperative setting is still sparse.

The published trials on ^177^Lu-PSMA-617 report a generally manageable safety profile, though adverse events varied by study and comparator. In the Vision trial, ^177^Lu-PSMA-617 was associated with higher rates of grade 3–4 hematotoxic adverse events including anemia in 13% of patients and thrombocytopenia in 8% compared to the SOC [10]. In TheraP, where mCRPC patients in the control arm received cabazitaxel, RLT with ^177^Lu-PSMA-617 was reported to have fewer adverse events with lower rates of neutropenia and fatigue [30]. Other frequently reported adverse events are xerostomia and mild hematologic toxicities. The sequential use of ^177^Lu-PSMA-617 followed by docetaxel in UpFrontPSMA similarly led to hematologic toxicities, without unexpected safety concerns [32]. Overall, ^177^Lu-PSMA-617 seems to lead to fewer severe side effects than taxane-based therapies especially in pretreated patients, making it an often better-tolerated option in the mCRPC setting.

Table 2 gives an overview about the studies with the highest impact on ^177^Lu-PSMA-RLT.

**Table 2:** Selected clinical phase II and III trials on ^177^Lu-PSMA-RLT in different disease settings

Study	Authors	Publication year	Patient cohort	Comparator	Main outcome
VISION [10]	Sartor et al.	2021	mCRPC, previous ARTA and taxane based regimens	SOC	*rPFS*: 8.7 vs. 3.4 months (*p* < 0.001; HR 0.40; 99.2% CI: 0.29–0.57) *OS:* 15.3 vs. 11.3 months (*p* < 0.001; HR 0.62; 95% CI: 0.5–0.74)
TheraP [30]	Hofman et al.	2021	mCRPC, post docetaxel, suitable for cabazitaxel	Cabazitaxel	*PSA reduction of >* *50%: *66 vs. 37 PSA responses *OS:* 19.1 vs. 19.6 months (HR: 0.97, 95% CI: 0.7–1.4 (*p* = 0.99))
PSMAfore [31]	Morris et al.	2024	mCRPC, post ARTA	ARTA switch	*rPFS* 11.60 months (95% CI 9.30–14.19) versus 5.59 months (4.21–5.95) (HR 0.49 [95% CI 0.39–0.61])
ENZA‑P [35]	Emmet et al.	2024	mCRPC, after ARTA or docetaxel	Enzalutamide vs. Enzalutamide + ^177^Lu-PSMA-617	*PSA-PFS* 13.0 months (95% CI 11.0–17.0) enzalutamide +^177^Lu-PSMA-617 group vs. 7.8 months (95% CI 4.3–11.0) enzalutamide group (HR 0.43, 95% CI 0.29–0.63, *p* < 0.0001)
UpFrontPSMA [32]	Azad et al.	2024	mCSPC	2 cycles of ^177^Lu-PSMA-617 followed by 6 cycles of docetaxel vs. 6 cycles docetaxel	*Undetectable PSA* (≤ 0.2 ng/mL) at week 48: 41% treatment arm vs. 16% comparator (OR 3.88, 95% CI 1.61–9.38; *p* = 0.0020)
LuTectomy [33]	Eapen et al.	2024	High-risk localized PCa	Single arm: neoadjuvant 2 cycles ^177^Lu-PSMA-617 prior to radical prostatectomy	*Tumor radiation absorption:* after cycle 1 for all lesions 35.5 Gy (19.5–50.1), with 19.6 Gy (11.3–48.4) delivered to the prostate *secondary*: 45% patients achieved > 50% PSA decline
PSMAAddition	Not yet published	Awaited 2025	mCSPC	^177^Lu-PSMA-617 + SOC vs. SOC alone	*Primary endpoint PFS, final analysis awaited*

*mCRPC* metastatic castration resistant prostate cancer, *ARTA* androgen receptor targeted agents, *mCSPC* metastasized castration sensitive prostate cancer, *PCa* prostate cancer, *SOC* standard of care, *ADT* androgen deprivation therapy, *rPFS* radiographic progression free survival, *HR* hazard ratio, *CI* confidence interval, *OS* overall survival, *OR* odds ratio

## Alternative radiopharmaceuticals for PSMA-radioligand therapy

Currently, ^177^Lu-PSMA-617 has an official registration status under the trade name Pluvicto® (Novartis, Irland), but some centers also use either commercially available or in-house produced ^177^Lu-PSMA agents.

In a matched-pair analysis of mCRPC patients treated with either PSMA-617 or PSMA-I&T both agents demonstrated comparable safety and efficacy profiles. The study involved 110 patients from two centers, with each group receiving at least two cycles of therapy. Toxicity assessments showed low rates of clinically relevant toxicities, with only 1 patient (1.8%) treated with ^177^Lu-PSMA-I&T experiencing grade III anemia, compared to 5 (9.1%) with ^177^Lu-PSMA-617, and 1 (1.9%) with grade III thrombopenia for the latter. No significant differences in OS were observed, with median OS of 12 months for ^177^Lu-PSMA-I&T and 13 months for ^177^Lu-PSMA-617. This study suggests that both radiopharmaceuticals are similarly effective and safe for treating mCRPC [34]. Yet, only ^177^Lu-PSMA-617 has an official drug registration status.

## Practical implementation of the PSMA-RLT and interdisciplinary procedure

As with other tumor entities, the decision to treat patients with PCa is carried out for each individual case in an interdisciplinary tumor board in which ideally urologists, oncologists, radiotherapists, radiologists, and nuclear medicine specialists participate.

The interdisciplinary treatment guidelines are summarized in Table 1.

Following documents should be available for this board: histology report of PCa, evidence of mCRPC, detection of sufficient PSMA expression of the tumor manifestations by PSMA-PET/CT analogue to the VISION study [10], white blood cells > 2.5/nl, thrombocytes > 75/nl, GFR > 30 ml/min. Life expectancy should be > 3 months [36].

Special attention is needed in case of renal outflow obstruction and previous myelotoxic chemotherapy. At least, 6‑week interval to previous myelotoxic chemotherapy is recommended. Renal scintigraphy prior to RLT might be useful for assessing kidney function and exclusion of a functional relevant urinary tract obstruction.

Radiopharmaceuticals are only received and administered by licensed staff in designated facilities in accordance with national regulations. According to national legislation in some countries, a medical physicist should be available for consultation.

There is an ongoing discussion whether dual-tracer imaging using ^18^F‑FDG-PET/CT in addition to the obligatory PSMA-PET/CT optimizes patient selection and treatment planning. ^18^F‑FDG PET/CT may offer more insights into prostate cancer biology. Studies using both tracers revealed interlesional heterogeneity with increasing detection of ^18^F‑FDG-positive tumor lesions among patients after each additional systemic treatment regimen. PSMA/FDG concordant lesions and particularly PSMA-negative and ^18^F‑FDG-positive lesions might affect treatment response, yet studies show primarily insights for risk stratification [37, 38].

Further studies are needed to establish dual-tracer imaging for improved treatment selection.

## Treatment plan

Any treatment plan can and should, however, be tailored to the individual patient needs as well as institutional protocols. In any case, the therapeutic department always needs to adhere to the local regulatory requirements and radiation safety practices. According to the current guideline of the EANM and SNMMI a standard treatment of 7.4 GBq ^177^Lu-PSMA-RLT is given intravenously every 6 weeks (±1 week) up to a maximum of 6 cycles, depending on efficacy and tolerability.

A prophylactic antiemetic therapy using, for example, a 5-HT3-antagonist can be given as needed. In addition, hydration should be kept in mind, which can be administered either orally or intravenously (e.g., 500–1000 ml isotonic saline or any other isotonic electrolyte solution). The patient should be encouraged to increase his fluid intake and void his bladder frequently for the first 3–4 days following treatment.

A posttreatment whole-body scintigraphy should be performed at least 2 h after treatment injection to confirm proper biodistribution and to rule out an extravasation [36].

If dosimetric calculations are performed, images at multiple time points postinjection are necessary [39, 40].

Patients have to be informed concerning the activity of lutetium-177 administered and the care required to minimize exposure of other people especially after hospital discharge, as well as the way to manage any radioactive waste, as described later.

## Predictive biomarkers and disease monitoring

Biomarkers may help in identifying early responses to treatment outcomes and selecting the patients, who might most likely benefit from a particular treatment.

As mentioned, PSMA-PET/CT is the basis in evaluation before starting RLT. Interim PSMA-PET/CT based response evaluation at 8–10 weeks after the second cycle of RLT has been shown predictive of overall survival and progressive disease in patients treated with ^177^Lu-PSMA-617 [23]. Additionally, prostate-specific antigen (PSA) response seems also reliable 2–3 weeks after the second cycle of therapy [41].

Results of a multicenter study indicated, that a PSA decrease of ≥ 30% after the first two therapy cycles is an early indicator of response to treatment and can be used in personalizing treatments for patients [42].

Rising PSA levels might indicate a lack of efficacy of ^177^Lu-PMSA-RLT. However, in a retrospective analysis a team of a bicentric study showed that patients with fluctuating PSA levels also have a survival benefit similar to those with constant PSA decline in contrast to those with continuously rising PSA levels [43].

**Fig. 2:**
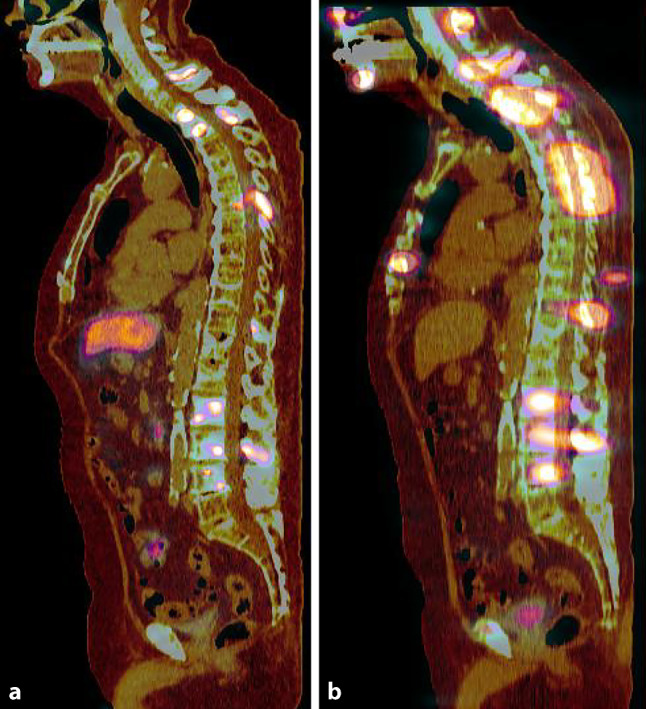
Images of an 83 year-old man with metastatic castration-resistant prostate cancer: ^18^F‑PSMA-PET/CT (**a**) and ^177^Lu-PSMA-RLT (**b**) posttherapeutic SPECT/CT (sagittal slice): several foci of PSMA-avid lesions can be seen on both the PET/CT and SPECT/CT images

A whole body planar and optionally SPECT/CT imaging on a gamma camera after each therapeutic administration is recommended (Fig. 2).

**Fig. 3:**
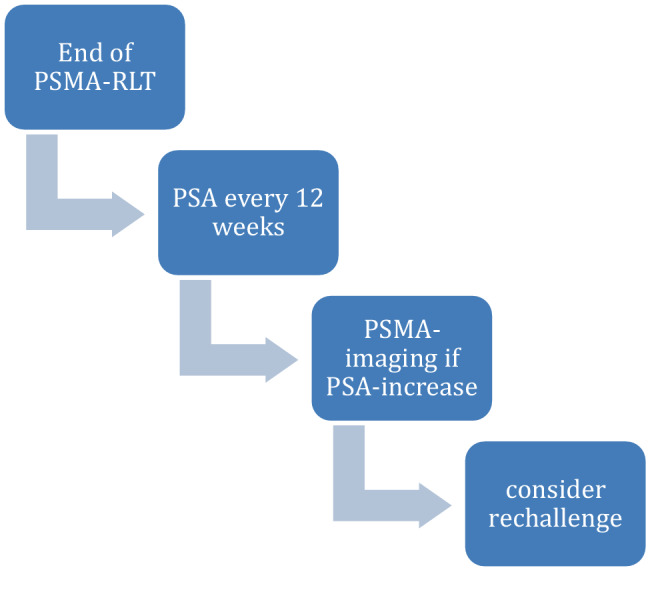
Follow-up after completion of ^177^Lu-PSMA-RLT

In the follow-up period after RLT, PSA measurements are recommended in general every 12 weeks. In case of a significant raise, additional imaging using again a PSMA PET/CT an be required (Table 3; Fig. 3).

**Table 3:** PSMA-RLT algorithm at a glance

Before treatment	Know the histology and treatment history of the patient. As for now, patients who are in mCRPC stage are candidates for treatment, yet changes in indications and recommendations for PSMA treatment may occur
	Proof of proper PSMA expression using a PSMA-PET/CT
	Proof of sufficient kidney and bone marrow function
	Know the patient’s performance status and individual needs concerning legally obligatory radioprotective measures
	Discuss the patient’s case in an interdisciplinary board
During treatment	Administer 4–6 cycles of ^177^Lu-PSMA-RLT every 6 weeks following guidelines and national as well as international regulations
	Perform posttherapeutic scintigraphy to assure proper quality of the therapeutic agent and to rule out extravasation
	Check kidney and bone marrow function regularly (e.g., prior to every treatment and 3 weeks thereafter)
	Use supportive care to minimize any discomfort of the mCRPC patient. There are no contraindications especially for using pain medication or any antianemic therapy
	Check PSA levels, but keep in mind that especially fluctuating levels are not an imperative reason to end treatment early
	If there is clinical suspicion for disease progression, perform a PSMA-PET/CT and discuss the patient in an interdisciplinary board
After treatment	If by any chance not done during treatment phase, re-establish regular uro-oncological visits
	Check PSA levels about every 12 weeks
	In case of clinical or biochemical progression, perform imaging, e.g. with a PSMA-PET/CT
	Discuss the patient’s case in an interdisciplinary board
	If a patient had a treatment benefit from previous RLT, still has ad adequate PSMA expression in the PET scan and still has an adequate performance status, rechallenge PSMA therapy may be a treatment option

*mCRPC* metastatic castration-resistant prostate cancer, *PSMA* Prostate-specific membrane antigen

Qualitative assessment criteria for PSMA PET/CT (RECIP 1.0) in mCRPC patients have been developed, which we believe are more suitable for daily practice than software-based quantitative assessment [44]. A summary of these criteria is provided in Table 4.

**Table 4:** Response evaluation criteria by follow-up PSMA-PET/CT, RECIP 1.0 [32]

Complete response	Partial response	Progressive disease	Stable disease
Absence of any PSMA uptake	≥ 30% decrease in PSMA tumor volume, without new lesion(s)	≥ 20% increase in PSMA tumor volume with new lesions	All other cases (including cases with new lesions but without significant change of tumor volume)

*PSMA* Prostate-specific membrane antigen

## Possibility of rechallenge

Rechallenge treatment with ^177^Lu-PSMA-RLT could be considered as a viable option for patients with mCRPC who have previously benefited from initial RLT yet experienced disease progression after a period of (partial) remission or disease stability.

Evidence from two retrospective patient cohorts, one using ^177^Lu-PSMA-617 [45], one using ^177^Lu-PSMA-I&T [46] as well as a prospective register study using ^177^Lu-PSMA-617 [47] support the possible efficacy and safety.

PSA response rates (> 50% decline) of 57–73% have been observed [45–47]. Overall survival of 22.7–25 months from the start of rechallenge treatment has been demonstrated [45, 47]. In a prospective registry multiple rechallenge series (up to 3) have shown continued efficacy in responding patients [47].

In all three studies the safety profile is favorable and comparable to the initial treatment cycles. Most adverse events are mild to moderate (grade 1–2) and no life-threatening (grade 4) events have been reported [45–47]. A slight increase in grade 3 hematological toxicities may occur but still remain manageable [45, 46].

Based on those findings a PSMA-RLT rechallenge for mCRPC patients could be considered if an initial response to the first PSMA-RLT has been demonstrated, alternative treatment options are limited, a sufficient PSMA expression is still detectable via PSMA-PET scan and the patient experiences disease progression after a period of remission. We strongly recommend discussing the indication for a rechallenge treatment in a multidisciplinary tumor conference. Careful patient selection, monitoring, and individualized treatment planning are essential to optimize outcome and minimize toxicity.

## Radiation protection measures

Taking into account the general radiation protection measures, the radiation exposure for hospital staff will be very low. Even in dialysis patients receiving this therapy, a low radiation exposure was found. Huang et al. demonstrated a low radiation exposure of 2–19 uSv after 20 h of injection of 3.1 GBq ^177^Lu-PSMA-617 in medical staff involved in hemodialysis of a patient with PCa [48].

Particular attention should be paid to waste created by patients with incontinence. If the therapy is performed on an outpatient basis, the waste (e.g., diaper pants) must be stored for up to 10 half-lives of lutetium-177, i.e., approximately 2 months, in a place where humans or animals are not expected to be present, such as a basement or an unused storage room, and only after this period added to the inactive waste. Outpatients should remain in the therapeutic facility, yet as isolated as possible, for at least 5 h to monitor side effects and complete the first excretion phase before discharge.

All patients must be instructed verbally and in writing on how to minimize exposure to others in accordance with Austrian law and radioprotection standards.

According to Austrian regulations, the dose limit for persons working voluntarily and individually as comforters and caregivers and providing essential support to patients is set at 3 mSv per year, provided that a minimum distance of 2 m from the patient is maintained. In our experience, assuming 4–6 therapy cycles (ongoing study) per year, the limit of 3 mSv will not be exceeded.

## Outlook

In contrast to lutetium-177, the radionuclide actinium-225 is an alpha emitter (particle emitter) that emits helium nuclei during radioactive decay. If these helium nuclei hit other molecules, they cause significantly more damage than the electrons of lutetium-177. The so-called linear energy transfer of actinium-225 to the tissue is significantly higher than that of lutetium-177. This means that a higher biological effectiveness in terms of the tumoricidal effect is likely [37]. In addition, the significantly shorter range of alpha radiation compared to beta radiation is seen as an advantage, as it means that surrounding tissue can be better protected and the tumor can be treated more precisely. Analogous to ^177^Lu-PSMA-RLT, the therapeutic goal of ^225^Ac-PSMA-RLT is to inhibit/slow down the growth of prostate cancer cells, e.g., in bone and soft tissue metastases. If the treatment response is favorable, further treatment cycles are possible after consultation with a uro-oncologist. The indications for ^225^Ac-PSMA-RLT are, on the one hand, progression/treatment failure after several cycles of ^177^Lu-PSMA therapy [38] and, on the other hand, a pronounced diffuse distribution pattern of the osseous metastases. In this case, the application of ^177^Lu-PSMA-RLT is expected to result in significantly higher bone marrow toxicity than with ^225^Ac-PSMA-RLT due to its greater range. Since all established therapies have already been exhausted in these patients before the indication for ^177^Lu-PSMA therapy is made (ARTAs, chemotherapy, PARP inhibitors), ^225^Ac-PSMA-RLT is a promising option [39]. The patients already treated with the therapy showed a remarkable response with xerostomia being a common side-effect [39]. It is expected that this therapy will play a major role in the treatment of these patients in the future and may also be used earlier in the therapy regimen [37].

As mentioned above, ^177^Lu-PSMA-RLT is currently undergoing broad scientific examinations in several studies in earlier disease stages than mCRPC (Table 5).

PSMA diagnostics as well as PSMA-based RLT have already had tremendous influence on prostate cancer management and are going to furtherly shape diagnostic and treatment options for an increasing amount of PCa patients.

In fact, ^177^Lu-PSMA-RLT is additionally under investigation even in neoadjuvant and localized oligometastatic disease states. The studies are still small and experimental, yet they give further insights into the potential of this treatment especially for high-risk PCa patients. Table 5 gives an overview of recent projects.

**Table 5:** Overview about neoadjuvant and experimental 177-Lu-PSMA-RLT trials

Trial	Phase	Population	Design	Primary endpoint	Status
PRELUDE	II	High risk PCa	2 cycles ^177^Lu-PSMA-617 RLT → surgery	Pathologic downstaging	Enrolling
LUNAR	II	Oligometastatic CSPC	^177^Lu-PSMA-RLT + stereotactic body radiotherapy (SBRT) vs. SBRT	PFS	Enrolling
NEPI	I/II	High risk PCa	^177^Lu-PSMA-617 ± ipilimumab → surgery	Feasibility/CR rate	Enrolling
LUPUS	I/II	High risk PCa	Intra-arterial ^177^Lu-PSMA-617 → surgery	Safety	Early phase

## Conclusion

The integration of advanced systemic therapies such as radioligand therapy (RLT) with radiolabeled prostate-specific membrane antigen (PSMA) ligands into the prostate cancer treatment has improved clinical outcomes in patients with metastatic castration-resistant prostate cancer (mCRPC).

However, given the growing number of patients with mCRPC requiring RLT and the limited number of treatment beds, it is necessary to offer RLT to outpatients as well. Health care providers have to increase resources for this increasingly important treatment.
